# A Healable and Mechanically Enhanced Composite with Segregated Conductive Network Structure for High-Efficient Electromagnetic Interference Shielding

**DOI:** 10.1007/s40820-021-00693-5

**Published:** 2021-08-02

**Authors:** Ting Wang, Wei-Wei Kong, Wan-Cheng Yu, Jie-Feng Gao, Kun Dai, Ding-Xiang Yan, Zhong-Ming Li

**Affiliations:** 1 College of Polymer Science and Engineering, State Key Laboratory of Polymer Materials Engineering, Sichuan University, Chengdu, 610065 People’s Republic of China; 2 The College of Chemistry and Chemical Engineering, Yangzhou University, Yangzhou, 225009 People’s Republic of China; 3 School of Materials Science and Engineering, Zhengzhou University, Zhengzhou, 450001 People’s Republic of China; 4 School of Aeronautics and Astronautics, Sichuan University, Chengdu, 610065 People’s Republic of China

**Keywords:** Electrostatic attraction, Healable, EMI shielding, Diels–Alder reaction

## Abstract

- The cationic waterborne polyurethanes microspheres with Diels-Alder bonds were synthesized for the first time.
- The electrostatic attraction not only endows the composite with segregated structure to gain high electromagnetic-interference shielding effectiveness, but also greatly enhances mechanical properties.
- Efficient healing property was realized under heating environment.

**Abstract:**

It is still challenging for conductive polymer composite-based electromagnetic interference (EMI) shielding materials to achieve long-term stability while maintaining high EMI shielding effectiveness (EMI SE), especially undergoing external mechanical stimuli, such as scratches or large deformations. Herein, an electrostatic assembly strategy is adopted to design a healable and segregated carbon nanotube (CNT)/graphene oxide (GO)/polyurethane (PU) composite with excellent and reliable EMI SE, even bearing complex mechanical condition. The negatively charged CNT/GO hybrid is facilely adsorbed on the surface of positively charged PU microsphere to motivate formation of segregated conductive networks in CNT/GO/PU composite, establishing a high EMI SE of 52.7 dB at only 10 wt% CNT/GO loading. The Diels–Alder bonds in PU microsphere endow the CNT/GO/PU composite suffering three cutting/healing cycles with EMI SE retention up to 90%. Additionally, the electrostatic attraction between CNT/GO hybrid and PU microsphere helps to strong interfacial bonding in the composite, resulting in high tensile strength of 43.1 MPa and elongation at break of 626%. The healing efficiency of elongation at break achieves 95% when the composite endured three cutting/healing cycles. This work demonstrates a novel strategy for developing segregated EMI shielding composite with healable features and excellent mechanical performance and shows great potential in the durable and high precision electrical instruments. 
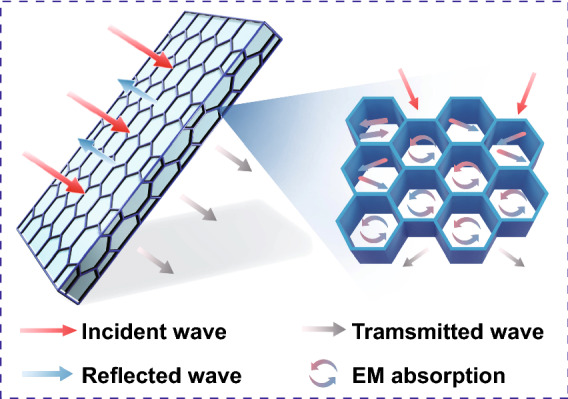

**Supplementary Information:**

The online version contains supplementary material available at 10.1007/s40820-021-00693-5.

## Introduction

With the development of electronics technology, especially the advent of 5G era, electromagnetic interference (EMI) shielding materials are increasingly required to prevent the electronic devices and human bodies from suffering intense electromagnetic radiation [1–3]. Considerable efforts have been devoted to fabricate conductive polymer composites (CPCs)-based EMI shielding materials due to the lightweight, easy processibility, low cost, corrosion resistance, etc. [4–6]. Normally, plenty of conductive fillers are necessary to obtain outstanding EMI SE in CPCs, which may result in low economic affordability, inferior flexibility and low processability [7–9]. Current researches mainly focus on structural design to improve conductive network to achieve high EMI SE [10–13]. Segregated structure, with conductive fillers confined located at interfaces of polymer regions, is considered to significantly increase the effective concentration of conductive fillers to construct perfect conductive network in CPCs [14–18]. Numerous works including our previous works have achieved outstanding EMI SE in CPCs with segregated structure [19–22], whereas the accompanying conductive filler–polymer region interfaces and interfacial crack would cause degraded mechanical properties. The simultaneous achievement of EMI SE and mechanical properties in segregated CPCs promises their great potential as advanced shielding materials for various applications including aerospace and smart electronic devices.

Over the practical situations, EMI shielding CPCs would inevitably generate micronotches or breaks when suffering external mechanical stimulus such as fatigue, abrasion and impact, which may result in performance degradation and even catastrophic failure of the devices [23–27]. Recently, many efforts have been done to develop healable EMI shielding CPCs with the polymer matrix owning reversible bonds [28]. For instance, a healable polyurethane with disulfide bonds was filled with carbon nanotube (CNT)/graphene oxide (GO)/MoS_2_/Fe_3_O_4_ nanoparticles, and the resultant composite exhibited healing efficiency of 70% in mechanical properties and nearly unaltered EMI SE post-healed for 24 h at room temperature [29]. Contacting the cracked polyacrylamide/cellulose nanofiber/CNT hydrogel for a week could recover the EMI SE with high retention of ~ 96% [30]. In the healing system, reversible Diels–Alder (DA) reaction is one such highly viable mechanism to achieve healing behavior in polymers ascribes to its simple process, minimal side reactions, highly efficient reversibility, moderate sensitivity to temperature and dynamic cross-linking structure [31]. It is worth noting that though considerable healable effects have been achieved, the reported EMI shielding CPCs mainly contained randomly distributed conductive fillers, while few works report healable CPCs containing segregated conductive networks. This is attributed to the challenged acquisition of polymer granules with healable features. The development of healable and segregated CPCs is of great significance to increase the lifespan and sustainability of high-performance EMI shielding devices. In our recent work, a healable polyurethane was made by external emulsification and used as a matrix to obtain the healable and segregated EMI shielding composite [32]. The composite at 7 wt% CNT content shows EMI SE of 41.2 dB and undesirable mechanical properties with stress of ~ 15.6 MPa and low elongation at break of ~ 340%, while the composite even cannot be formed when the CNT loading up to 10 wt%.

Herein, the cationic waterborne polyurethanes microspheres with DA bonds (denoted as CPA microspheres) were synthesized for the first time to adsorb anionic CNT/GO hybrid via electrostatic attraction to help the construction of segregated conductive network in the resultant CNT/GO/CPA composite. The healable EMI shielding composite was obtained by taking the advantage of electrostatic assembling between the negatively charged carbon nanotube/graphene oxide and positively charged CPA. The electrostatic attraction not only endows the composite with segregated structure to gain high EMI SE, but also greatly enhances mechanical properties. The composite with 7 wt% filler loading owns high EMI SE of 46.0 dB and excellent mechanical property with stress of ~ 32.5 MPa and elongation at break of ~ 431%. And the composite still exhibits sufficient flexibility even at 10 wt% filler loading with elongation at break up to ~ 400%. Moreover, the composite could recover the EMI shielding and mechanical performances on subjecting to the severe mechanical damages due to the DA bonds. The CNT/GO/CPA composites could be repeatedly healed with 90% retention in EMI SE and 95% healing efficiency in mechanical properties. Such a unique CNT/GO/CPA composite helps to improve the long-term stability of EMI shielding materials in actual application.

## Experimental Section

### Materials

CNTs (NC 7000 series) with surface area of 250–300 m^2^ g^−1^, average diameter of 9.5 nm, average length of 1.5 µm were supplied by Nanocyl S.A., Belgium. Isophorone diisocyanate (IPDI), glycerol 1,2-carbonate, furfurylamine, dibutyltin dilaurate (DBTDL), 1,4-butanediol (BDO) and 1,1′-(methylene-di-4,1-phenylene)-bismaleimide (BMI) were purchased from Aladdin, China. The chemicals were used for subsequent reactions without any treatment. Polytetramethylene ether glycol (PTMG) (Mn = 2000 g mol^−1^) was obtained from Aladdin and placed in a vacuum oven at 110 ℃ for 2 h to remove water prior use. Methyldiethanolamine (MDEA) and Methyl ethyl ketone (MEK) were provided by Kelong Chemical Co., Ltd., China.

### Synthesis of Cationic Waterborne Polyurethane with DA Bonds (CPA)

The synthesis process of CPA is shown in Fig. S1. Glycerol 1,2-carbonate (4.28 mL) and furfurylamine (4.46 mL) were mixed in a round-bottom flask. The mixture was stirred and heated at 60 ℃ for 3 h in an oil bath to obtain the Diol. Then Diol (0.65 g), IPDI (7.5 g), PTMG (10.0 g) were placed in the round-bottom flask. After the addition of 5 µL DBTDL and a moderate amount of MEK, the solution was heated at 60 ℃ for 2 h with homogeneous stir. 1.4 g MDEA was diluted with MEK and added dropwise into the above mixture. Subsequently, the mixture was reacted for 2 h to get the -NCO groups terminated oligomer. To extend the intermediated products, a precalculated amount of BDO (0.5 g) was added slowly to the reaction solution. After 1 h of chain extension reaction, BMI (1.5 g) was added and fully dissolved in the solution and reacted for another 6 h to generate DA bonds. During the reaction process, MEK was added occasionally to prevent excessive viscosity of the reaction solution. After that, the reaction product and acetic acid (0.8 g) were mixed at 40 ℃ for 30 min with continuous stirring, which was aimed to neutralize the ionic centers of MDEA. To get the CPA emulsion, the deionized water was added to the obtained mixture under vigorous stirring for 1 h. Finally, the MEK was removed with rotary evaporator. The ultimate CPA emulsion was cream yellow with a solid content of 25 wt%.

### Preparation of the CNT/GO/CPA Composite

**Fig. 1:**
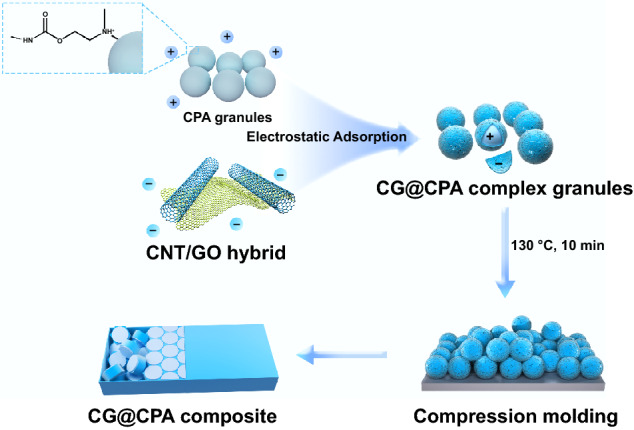
Schematic diagram of the fabrication process for CG@CPA composite

The fabrication process of the CNT/GO**/**CPA composite is schematically illustrated in Fig. 1 The aqueous suspension of CNT/GO (GO/CNT = 1:5) with negative charge was firstly prepared. Then, the CNT/GO suspension was quantitatively added into the positively charged CPA emulsion with vigorous stirring. The CNT/GO/CPA coagulation was obtained by electrostatic self-assembly, following with suction filtration and freeze-dried process to get the CNT/GO/CPA complex granules. The CNT/GO/CPA composite was fabricated by compression molding of the CNT/GO/CPA complex granules at 130 ℃ for 10 min. The ultimate composites were named as CG@CPA-x, where x represents the weight content of CNT/GO hybrid in the composite. The bulk CPA film was also prepared for comparison, under the same condition without the addition of CNT/GO hybrid.

### Characterization

Zeta potential measurements were determined with a Zetasizer Nano ZS90 instrument (Malvern, UK). The size distribution of CPA emulsion was measured using Mastersizer 2000 laser particle size analyzer. The existence of quaternary ammonium salt in CPA microspheres was proved by an X-ray photoelectron spectroscopy (XPS, XSAM800, Shimadzu-Kratos Ltd., Japan). Fourier-transform infrared (FTIR) spectroscopy (Nicolet 6700, Thermal Scientific, USA) was used to record the chemical structure of the composite. The electrical conductivities of the composites were conducted on an RTS-9 four-point probe meter (Guangzhou Four-Point Probe Technology Co., Ltd., China). Mechanical properties were measured on a universal tester (Model 5576, Instron Instruments, USA) with a crosshead speed of 50 mm min^−1^ and gauge length of 20 mm. At least five dumbbell-shaped specimens with 4.0 mm width and 50.0 mm length were tested for each group. Optical microscope (OM, Olympus Co., Tokyo, Japan) equipped with a heating stage (CSS450, Linkam Scientific instruments, UK) was used to monitor the healing process of the crack among the specimen. The temperature rose to 130 °C for 5 min with a heating rate of 10 °C min^−1^. And taking micrographs to record the healing process with time intervals of 30 s. Morphology and microstructures were observed with a scanning electron microscope (SEM; Inspect-F, FEI, USA) at an acceleration voltage of 5 kV and a FEI Tecnai F20 transmission electron microscope (TEM) at an acceleration voltage of 200 kV. For SEM observation, the specimens were soaked into the liquid nitrogen for 30 min and cryo-fractured. All of the specimens were coated a thin layer of gold before SEM test. For TEM observation, CG@CPA composite specimens were cryo-microtomed using a Leica EM UC6 equipment to get the ultrathin cryo-sections of 50 to 100 nm thickness, which were collected and directly supported on a copper grid. The circular samples with 12.0 mm diameter and 2.0 mm thickness were used to test EMI SE on an Agilent N5230 vector network analyzer within 8.2–12.4 GHz. And the detailed calculate formulas of SE_T_, SE_R_ and SE_A_ were shown in Supporting Information (SI).

## Results and Discussion

### Structure and Composition Characteristics

**Fig. 2:**
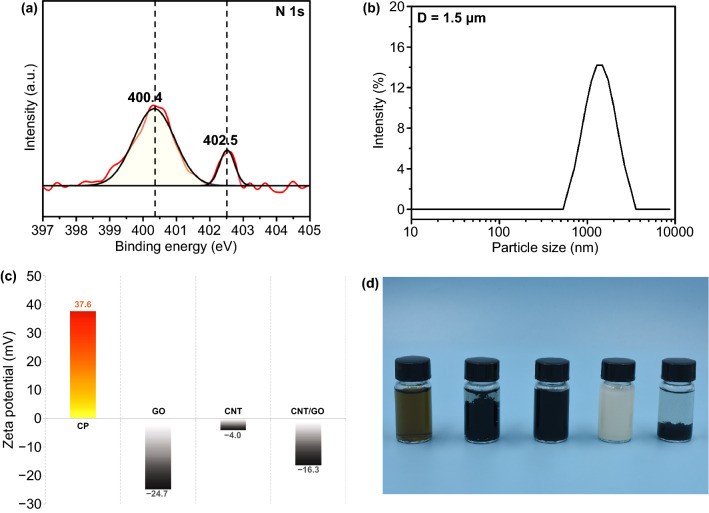
**a** XPS survey spectra of CPA. **b** Size and distribution of CPA particles. **c** Zeta potential of CPA, GO, CNT and CNT/GO aqueous dispersions. **d** Digital picture of GO, CNT, CNT/GO, CPA and CG@CPA aqueous dispersions (from left to right in the picture) after being placed for 12 h

The quaternary ammonium ions in the CPA are necessary for the electrostatic self-assembly. To demonstrate the existence of quaternary ammonium ions, XPS spectra of CPA were carried out, as shown in Fig. 2a. In the N 1* s* spectrum, two peaks at 400.4 and 402.5 eV emerged which are related to C–N bond of urethane and cationic –NH(CH_3_)_2_^+^ from MDEA molecular chain respectively, indicating the successful preparation of cationic waterborne polyurethane. The FTIR spectroscopy (Fig. S2) further demonstrates the chemical structure of the CPA and relevant statement is shown in SI. The average size of CPA microspheres was 1.5 µm (Fig. 2b). The Zeta potential test can not only measure the possibility of electrostatic adsorption, but also evaluate the stability of the dispersion. MDEA converted to quaternary ammonium salt on the CPA molecular chain through acid–base neutralization reaction, endowing the CPA microspheres with the Zeta potential value of + 37.6 mV (Fig. 2c). The higher absolute value of Zeta potential illustrates that abundant electrostatic repulsive energy is presented on the surface of CPA specimens, demonstrating better physical stability [33]. The Zeta potential values for the GO, CNT, CNT/GO aqueous dispersion are − 24.7, − 4.0, − 16.3, respectively, indicating that a small amount of GO can help CNT to be uniformly dispersed in water due to the *π*–*π* conjugation between them [34–36]. The CNT/GO dispersion was added into the CPA emulsion with homogeneous stirred. The positive charged surfaces of the CPA particles were partly neutralized by the negative charge of the hybrid filler, thus the potential drops. Figure 2d shows the dispersion condition of the GO, CNT, CNT/GO hybrid, CPA microspheres and CG@CPA complex in deionized water (from left to right) after settled for 12 h. Significantly, the CNT/GO are continuously assembled on the CPA microspheres by electrostatic attraction and further settle completely.

**Fig. 3:**
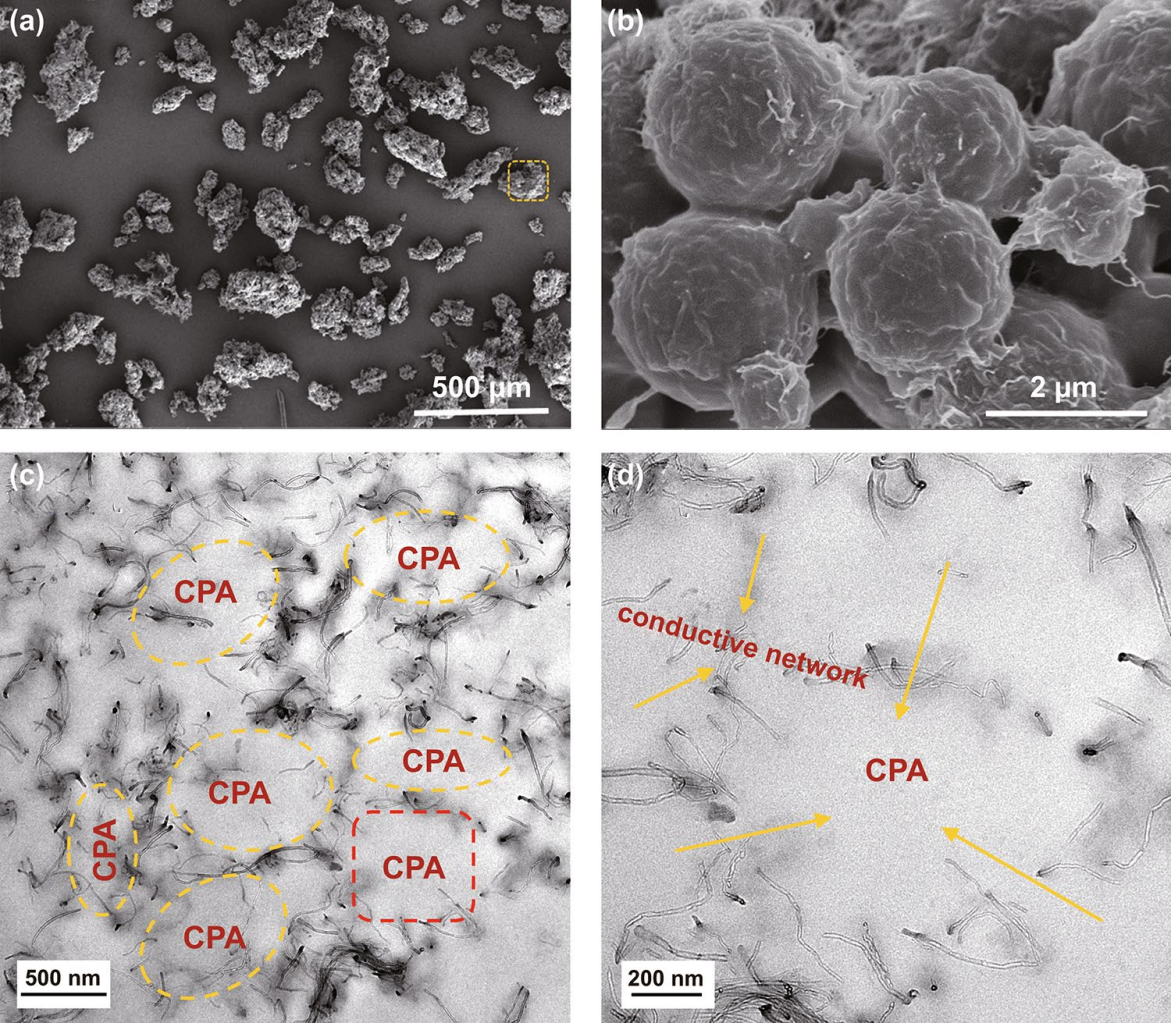
**a, b** SEM images of the CG@CPA-3 particles. **c, d** TEM images of the CG@CPA-3 composite

Figure 3a shows that the CG@CPA powder is composed by numerous spherical complex granules. In the magnified SEM image (Fig. 3b), numerous CNT/GO hybrid are compactly wrapped on the surface of the CPA granules, which is conducive to the construction of efficient conductive networks in the final CG@CPA composites. The CG@CPA powder was further hot-pressed to prepare CG@CPA composites, and the internal morphology is shown in Fig. 3c, d. As expected, conductive fillers are all selectively located at the interfaces of the CPA domain, forming a well-defined and interconnected network. Encouragingly, the unique 3D conductive network helps to improve the electrical and EMI shielding capacities.

### Electrical and EMI Shielding Performances of CG@CPA Composite

**Fig. 4:**
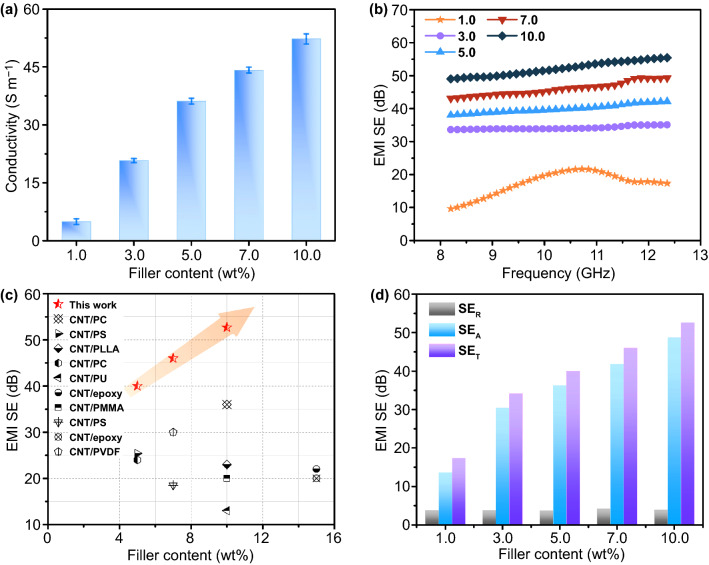
**a** Electrical conductivity **b** EMI SE of the CG@CPA composite with various CNT/GO content. **c** Comparison of EMI SE of CG@CPA composite and other reported composites **d** Comparison on SE_*T*_, SE_*A*_ and SE_*R*_ of CG@CPA composites

Figure 4a reveals the electrical conductivity of the CG@CPA composite. Obviously, the electrical conductivity improves with the increasing CNT/GO content. The CG@CPA-1 exhibits the conductivity of 5.0 S m^−1^, which already exceed the required value of commercial EMI shielding materials (1.0 S m^−1^) [37, 38]. The electrical conductivity of the CG@CPA-10 could attain to 52.0 S m^−1^ due to the highly efficient segregated conductive network. The desirable electrical conductivity is an essential prerequisite for the CG@CPA composite to obtain outstanding EMI shielding performance [39–41]. Figure 4b reveals EMI SE of CG@CPA composites in the frequency range of 8.2–12.4 GHz (X-band). As the CNT/GO content increases, the EMI SE of the composite apparently enhances, which is consistent with the trend of electrical conductivity. With higher CNT/GO loading, more free electrons will be generated to interact with the incoming EM waves and thus improve the EMI SE. CG@CPA-3 exhibits the EMI SE of 34.1 dB, far surpassing the target value of the commercially applicable EMI shielding materials (20 dB) [42]. CG@CPA-10 obtains the maximum EMI SE of 52.7 dB, which could block 99.9995% incident electromagnetic wave. The EMI SE (34.1 dB) of the CG@CPA-3 composite is also superior to 27.7 dB for our previously reported segregated CNT/PU composite by ball milling (for CNT@PUDA-3), indicating that the electrostatic attraction has an auxiliary effect on the perfection of the segregated conductive network. Moreover, the achieved high EMI SE in the CG@CPA composite is competitively compared with most previously reported composites, as summarized in Fig. 4c and Table S1 [43–52].

**Fig. 5:**
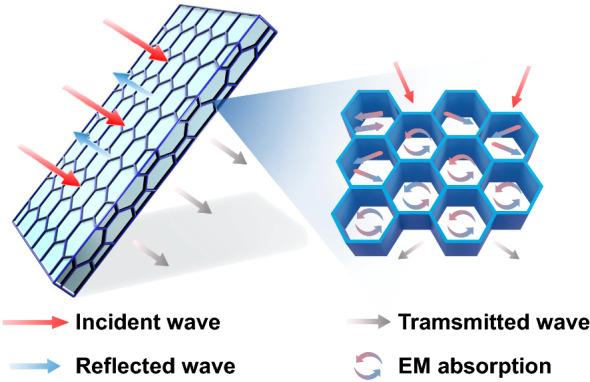
EMI shielding mechanism of the CG@CPA composite

To better clarify the shielding mechanism for the CG@CPA composite, SE_R_ and SE_A_ calculated from the measured scattering parameters are summarized in Fig. 4d. It can be obviously seen that SE_*A*_ shows almost the same variation tendency and contributes overwhelmingly with SE_*T*_ (EMI SE), whereas SE_*R*_ hardly changes, regardless of CNT/GO hybrid loading, demonstrating the absorption dominated EMI shielding mechanism. For instance, SE_*R*_, SE_*A*_ and SE_*T*_ of CG@CPA-10 are 4.0, 48.7 and 52.7 dB, manifesting that the SE_*A*_ makes a large proportion of 92% contribution to the SE_*T*_. The SE_*A*_ dominated mechanism mainly originated the well-defined segregated 3D conductive network (Fig. 5). The CNT/GO is located at the interfaces of the CPA domain to form a honeycomb-like EMI shielding cage. The CNT/GO conductive layer could generate mass interfaces to enhance reflect and scatter EM waves, thereby extending the propagation path of the EM waves and further attenuating electromagnetic energy. And the trapped EM waves could hardly escape from the compact core–shell structure before dissipate in the form of thermal energy.

### Mechanical Properties of the CG@CPA Composite

**Fig. 6:**
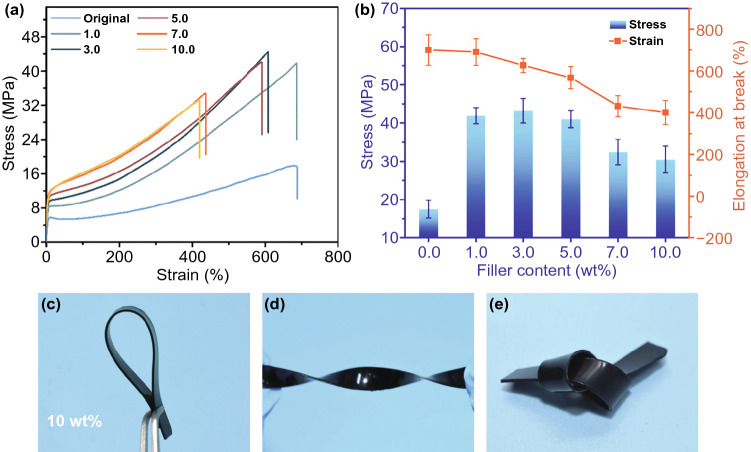
**a** Stress–strain curves and **b** average stress and elongation at break of CG@CPA composites with various CNT/GO hybrid loadings. **c–e** Photographs of the CG@CPA-10 with great flexibility

The successful construction of the segregated structure endows the CG@CPA composite with a high EMI SE. In addition, the CNT/GO hybrid firmly attached to the CPA particles through electrostatic attraction, forming the robust composite with enhanced interface bonding. The detailed quantified mechanical properties are shown in the stress–strain curves (Fig. 6a, b). The pure CPA represents satisfactory flexibility with a relatively low stress of ~ 17.5 MPa and an elongation at break (EB) of ~ 700%. As the CNT/GO content increases, the Young’s modulus of the CG@CPA composite gradually rises on account of the stiff hybrid filler inside the composite. Meanwhile, the stress rises sharply to ~ 43.1 MPa of CG@CPA-3 due to the efficient load transfer from the CPA to the compact CNT/GO network. Moreover, the CG@CPA composite still shows superior mechanical properties that surpass other reported composites with similar structures [53–55], especially for the CG@CPA-3 with high stress and high EB (~ 626%). And the CG@CPA-7 exhibits EB of ~ 431% and stress of ~ 32.5 MPa, which simultaneously increases the strength and EB compared to our previous CNT@PUDA-7 (340%, ~ 15.6 MPa). Besides, the robust CG@CPA-10 remains sufficient flexibility with EB of ~ 400% and stress of ~ 30.5 MPa, which is more than enough for the application in flexible electronic devices [56]. As shown in Fig. 6c–e, the CG@CPA-10 even can be arbitrarily bent or folded even, showing great flexibility while the CNT@PUDA-10 cannot be efficiently prepared. Obviously, the electrostatic attraction in the segregated CNT/GO/CPA composite makes the interfaces connection tighter and solves the interface defects between the matrix and the filler in conventional segregated CPCs, contributing to enhanced mechanical properties.

### Thermal-Driven Reversibility of CG@CPA Composite

**Fig. 7:**
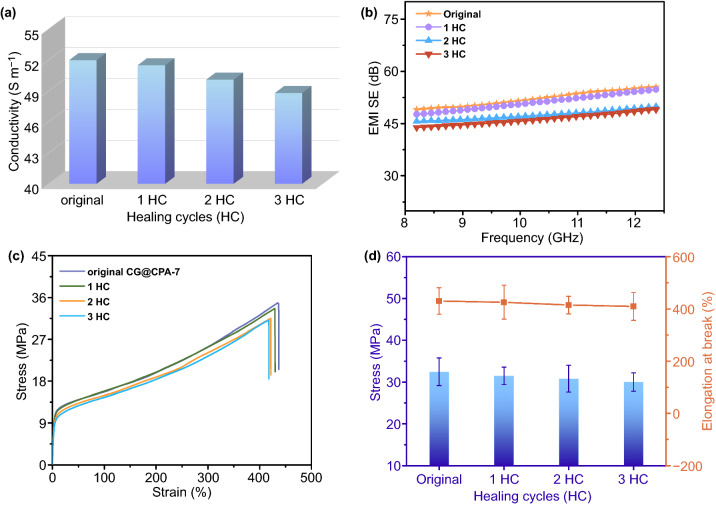
**a** Electrical conductivity and **b** EMI SE versus the healing cycles of the CG@CPA-10. **c** Stress–strain curves and **d** average stress and elongation at break of initial and healed CG@CPA-7

While having excellent EMI shielding and mechanical properties, the CG@CPA composite also owns healable function based on the DA bond. To test the healing behavior in terms of electrical and EMI shielding performances, original CG@CPA-10 was damaged into two halves. Then, the two parts were compacted together to treat at 130 ℃ for 10 min and then put at 60 ℃ for 2 h to measure the EMI SE again. The electrical conductivity and EMI SE varied with healing cycles are shown in Fig. 7a, b. For convenience, the first, second and third cutting/healing cycles of the CG@CPA-10 that occur at the same location are called 1 HC, 2 HC and 3 HC, respectively. The electrical conductivity of 1 HC is 51.5 S m^−1^, which is only 1.0% lower than the original value (52.0 S m^−1^). Encouragingly, electrical conductivity of 3 HC (~ 48.8 S m^−1^) still maintains a high retention of 94%, suggesting that CG@CPA composite possesses eminent repeatable repair capability. The EMI shielding performance of CG@CPA composite can also be repeatedly healed. For instance, the EMI SE of the 1 HC is 51.1 dB with only slight decreases compared to initial value (52.7 dB). And the recovery efficiency in EMI SE (the ratio of EMI SE before and after healing) of the 3 HC (47.3 dB) can keep 90%. From the above results, it can be inferred that the segregated conductive pathway at the fracture interfaces of the composite is well reconstructed after the repeated healing process, achieving high healing efficiencies on electrical and EMI shielding performances.

The repeatable repair ability of CG@CPA composite is also adapted to mechanical properties. As shown in Fig. S3, the CG@CPA-7 is cut in half. And it can lift a 50 g weight without fracture or regeneration of scars after healing process. The more intuitive healing process was recorded in Video S1. Put the fractures of the broken CG@CPA-7 together on a hot stage with 130 °C for 5 min and then continue to treat them at 60 °C for 2 min. The sample is healed after the above treatment and not broken when suffer from the tension of hands, which further illustrate the successful recovery of mechanical properties. To quantitatively evaluate the healing ability, the tensile measurement was used to test mechanical properties of repaired and the virgin specimens. The healing efficiencies were calculated by ratio of EB of the healed composites to the initial samples. The mechanical properties of the CG@CPA-7 with different cutting/healing cycles are shown in Fig. 7c, d. The mechanical curves of the 1 HC and the original sample basically coincide, indicating that the mechanical properties have been efficiently restored. Additionally, the composite can be healed repeatedly. For example, even 3 HC has a similar EB of ~ 410% compared to ~ 431% of the virgin one and displays a very close tensile strength (~ 30.0 MPa) to the initial value (32.5 MPa). The healing efficiencies for 1 HC, 2 HC and 3 HC are ~ 99%, ~ 96% and ~ 95%, respectively, demonstrating that CG@CPA composite can be repaired for multiple times with only a weak reduction in the healing efficiency. And the healing efficiencies of the EB are higher than the previously reported similar composite [26, 57–59]. The CG@CPA composite with superior and healable mechanical properties has significant potential to apply in the robust EMI shielding instrument.

**Fig. 8:**
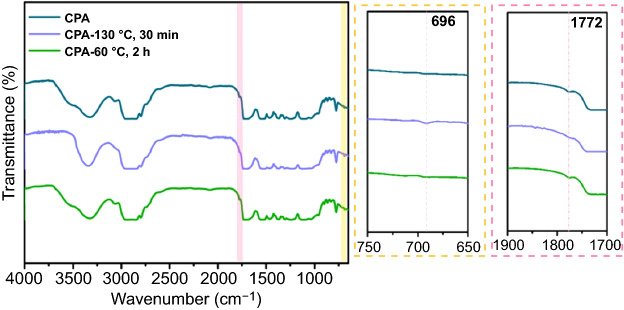
FTIR spectra of initial CPA, CPA-130 ℃ 30 min (refer the initial CPA after heating at 130 ℃ for 30 min, retro-DA) and CPA-60 ℃ 2 h (means the CPA-130 ℃ 30 min continue to place at 60 ℃ for 2 h to re-crosslinked due to DA reaction)

The reversibility of DA reaction in CPA was demonstrated through FTIR spectroscopy in terms of a heating/cooling procedure (Fig. 8). The characteristic peak at 1772 cm^−1^ corresponds to the DA adduct which is produced by the forward DA reaction. The peak of maleimide obtained through the reverse DA reaction of CPA at 130 ℃ should be located at 696 cm^−1^ [60]. The CPA curve appears the peak of 1772 cm^−1^ but no peak of 696 cm^−1^, showing that maleimide successfully participated in the DA reaction. Nevertheless, the peak of 1772 cm^−1^ disappeared and 696 cm^−1^ emerged when the original CPA was heat-treated at 130 °C for 10 min, which confirmed the occurrence of the retro-DA reaction. The retro-DA sample was processed at 60 ℃ for 2 h to achieve the successful reconnection of DA bonds by the maleimide and furan groups, which can be demonstrated by the distinctly appearance of the 1772 cm^−1^ and the disappearance of 696 cm^−1^. Overall, the reversibility of the DA/retro-DA reaction in CPA is fully demonstrated based on thermal stimulation. Further, the healing behavior of the CG@CPA composite was systematically investigated.

**Fig. 9:**
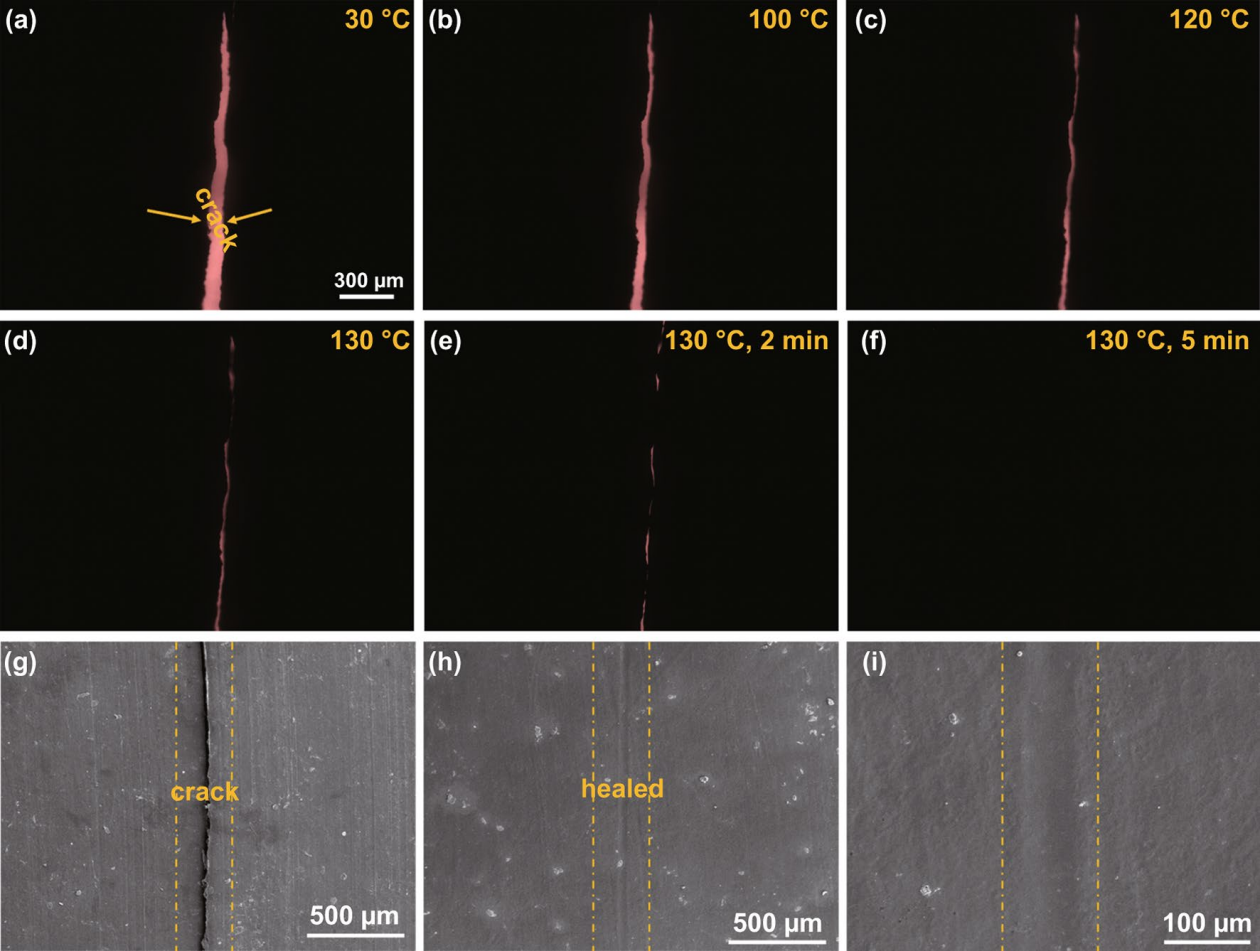
**a–f** OM images of the healing process at different temperatures of the crack (the composite is black, thus the transparent part <red> is the location of the crack) on the CG@CPA-7. **g–i** SEM images of the surface of the CG@CPA-7 before and after healed

The OM with a heat stage was used to record healing process of the CG@CPA-7. Firstly, an irregular crack with ~ 83 µm width was produced on the CG@CPA-7 by a blade. And then, the damaged composite was placed on the heating stage and heated from room temperature to 130 °C with a heating rate of 10 °C min^−1^. As exhibited in Fig. 9a–f, the crack shrinks slightly as the temperature arrives at 120 ℃ and almost diminished when the temperature maintains at 130 °C for 1 min. Ultimately, the crack closed completely at 130 °C for 5 min. More clearly, the SEM was used to observe the healing effect as shown in Fig. 9g–i. It is seen that the cracks of the sample were completely repaired with only minor scratches left, proving that the crack in CG@CPA composite can be healed quickly and completely due to the presence of DA bonds inside the system.

**Fig. 10:**
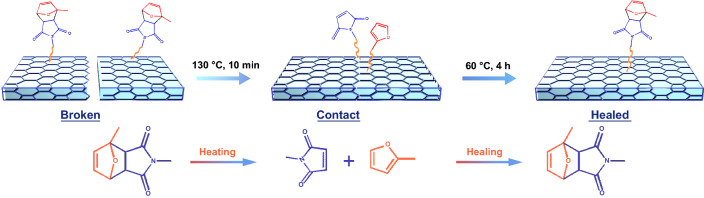
Healing mechanism of the CG@CPA composite based on the DA bonds

Further, the healing mechanism of the CG@CPA composite based on the DA bonds is analyzed, as shown in Fig. 10. The damaged CG@CPA sample undergoes heat treatment at 130 °C for 5 min. It is aim to guarantee that the molecular chain of CG@CPA composite to temporarily split into lower molecular weight substances via retro-DA reactions, thereby causing viscous movement of the cleaved fragments and the involved CNT/GO networks at the injured section to glue the broken sections together again. After the disappearance of cracks, the sample is maintained at 60 °C for 2 h, making sure the reconstruction of the DA bonds through DA reactions by the major dissociative maleimide and furan moieties. Finally, the cleaved molecular chains are re-cross-linked and the broken conductive networks are re-built, forming an intact CG@CPA composite with original mechanical and EMI shielding properties.

## Conclusions

A healable and durable EMI shielding composite was innovatively designed by introducing the CNT/GO hybrid onto a healable cationic waterborne polyurethane (CPA) via electrostatic assembly. The continuous segregated conduction network is formed with the CNT/GO hybrid that selectively distributes in the interfaces of CPA matrix, supplying outstanding EMI shielding performance. Additionally, the composite can be healed effectively on suffering the external mechanical stimulus due to the DA bonds in the CPA molecular chain. The EMI SE of CG@CPA-10 could attain to 52.7 dB and thermal-driven healed to 47.3 dB even undergo three cutting/healing cycles. Specifically, the interfaces interaction between the CPA and CNT/GO hybrid is enhanced by the electrostatic attraction, achieving excellent mechanical properties of the CG@CPA composite. For instance, CG@CPA-3 exhibits high stress of ~ 43.1 MPa and high EB of ~ 626%. And the mechanical properties can also be repaired with high healing efficiency of 95% even after three recovery cycles. This novel CG@CPA composite with stable and superb EMI shielding performance provides a convenient strategy for designing advanced electrical devices.

## Supplementary Information

Below is the link to the electronic supplementary material.

Supplementary file1 (PDF 373 KB)

Supplementary file2 (MP4 9200 KB)
