# Supplementary material for: A Healable and Mechanically Enhanced Composite with Segregated Conductive Network Structure for High-Efficient Electromagnetic Interference Shielding
Source: Nanomicro Lett. 2021 Aug 2;13:162. doi: 10.1007/s40820-021-00693-5 (PMC8329141; doi:10.1007/s40820-021-00693-5)
Supplement: Supplementary file 1 — Supplementary file1 (PDF 373 KB) [file 40820_2021_693_MOESM1_ESM.pdf]

Supporting Information for

A Healable and Mechanically Enhanced Composite with Segregated Conductive Network Structure for High-Efficient Electromagnetic Interference Shielding

Ting Wang[1], Wei-Wei Kong[1], Wan-Cheng Yu[1], Jie-Feng Gao[2], Kun Dai[3], Ding-Xiang Yan[1, 4, *], Zhong-Ming Li[1, *]

[1]College of Polymer Science and Engineering, State Key Laboratory of Polymer Materials Engineering, Sichuan University, Chengdu 610065, P. R. China

[2]The College of Chemistry and Chemical Engineering, Yangzhou University, Yangzhou 225009, P. R. China

[3]School of Materials Science and Engineering, Zhengzhou University, Zhengzhou 450001, P. R. China

[4]School of Aeronautics and Astronautics, Sichuan University, Chengdu, 610065, P. R. China

[*]Corresponding authors. E-mail: yandingxiang@scu.edu.cn (Ding-Xiang Yan), zmli@scu.edu.cn (Zhong-Ming Li)

S1 Synthesis of the CPA

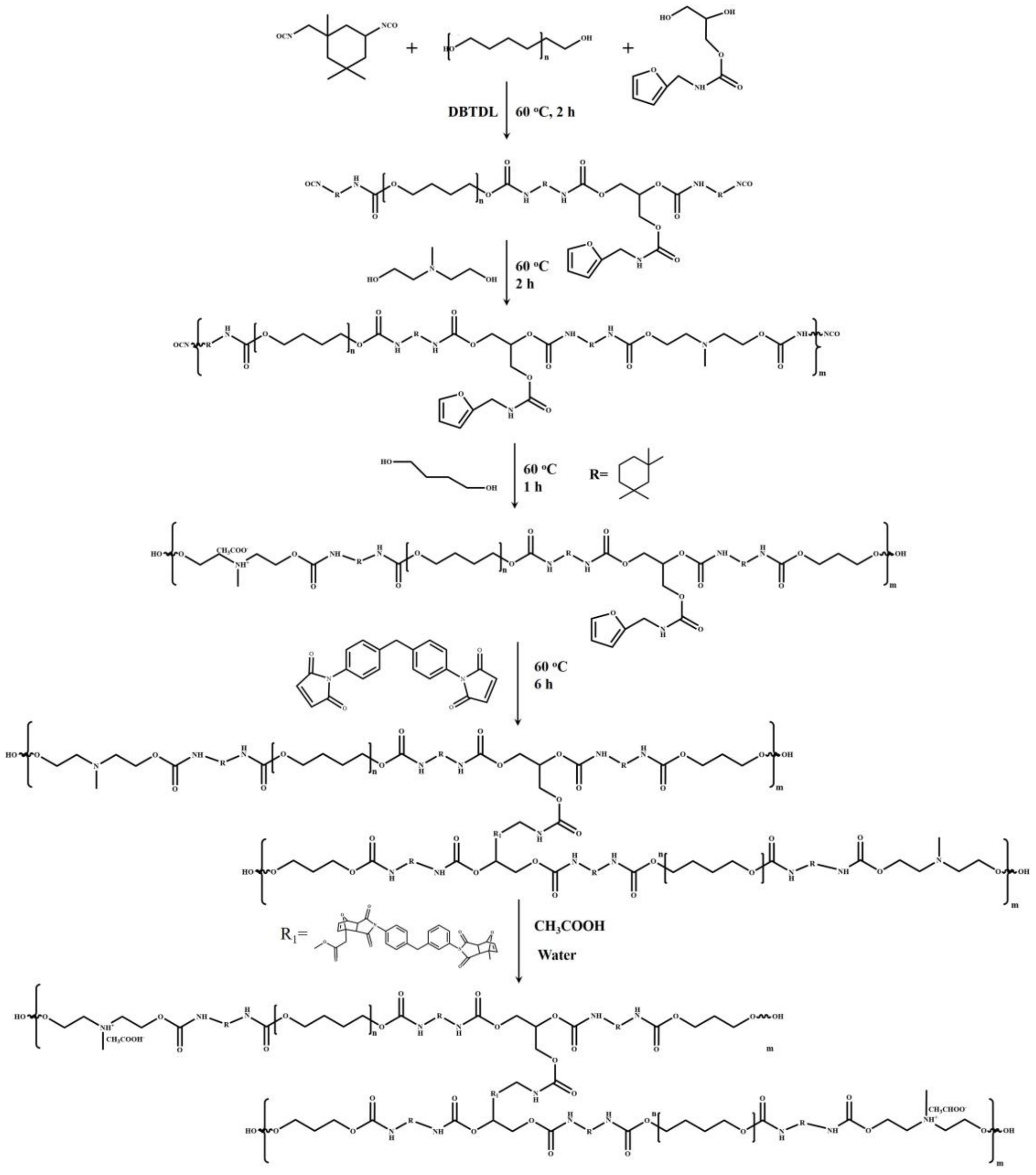

Fig. S1 Synthesis of CPA emulsions

S2 Calculation of EMI SE

EMI SE (SE_T), SE_R, and SE_A was obtained by the recorded scattering parameters (S_{11} and S_{21}). Then reflected power (R), transmitted power (T), and absorbed power (A), EMI SE (SE_T), microwave reflection (SE_R), and microwave absorption (SE_A) were calculated using the equations as follows.

$$R = |S_{11}|^2 \qquad \text{(S1)}$$

$$T = |S_{21}|^2 \qquad (S2)$$

$$A = 1 - R - T \qquad (S3)$$

$$SE_R = -10\lg(1-R) \qquad (S4)$$

$$SE_A = -10\lg\left(\frac{T}{1-R}\right) \qquad (S5)$$

$$SE_T = SE_R + SE_A + SE_M \qquad (S6)$$

When EMI SE is higher than 10 dB, microwave multiple internal reflections (SE_M) are neglectable [S1, S2].

S3 FTIR Characterization of the CPA

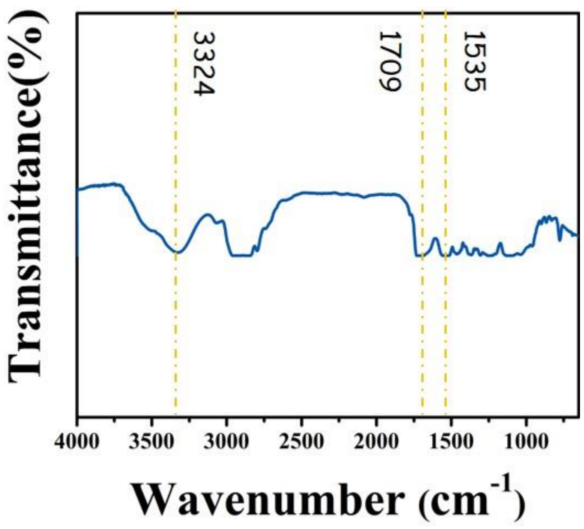

Fig. S2 FTIR spectra of CPA

The peaks at 3324 and 1535 cm^{-1} related to the urethane N-H stretching and deformation vibration, respectively. The characteristic peak at 1709 cm^{-1} is assigned to the C=O of urethane. Besides, the adsorption band of –NCO at 2270 cm^{-1} do not appear, indicating the complete conversion of IPDI to urethane. Furthermore, the weak absorption band at 1772 cm^{-1} corresponds to the DA adducts, manifesting that the DA bond was successfully incorporated into CPA chain. The above special peaks prove the successful synthesis of waterborne polyurethane with DA bond.

S4 Comparison of EMI SE of CG@CPA Composite with the Reported Literature

Table S1 Comparison of EMI SE for our CG@CPA composite with various CNT/polymer composites

Conductive filler	Content (wt%)	Thickness (mm)	EMI SE (dB)	Refs.
CG@CPA	5	2.0	40	this work
CG@CPA	7	2.0	46	this work
CG@CPA	10	2.0	52.7	this work
CNT/PC	10	2.0	36	[S3]
CNT/PS	5	2.0	25.4	[S4]
CNT/PLLA	10	2.5	23	[S5]
CNT/PC	5	2.0	24	[S6]
CNT/PU	10	2.0	13	[S7]
CNT/epoxy	15	2.0	22	[S8]
CNT/PMMA	10	4.5	20	[S9]
CNT/PS	7	1.2	18.5	[S10]
CNT/epoxy	15	1.5	20	[S11]
CNT/PVDF	7	2	30	[S12]

[a]PC, PS, PLLA, PMMA, PVDF are polycarbonate, polystyrene, poly (l-lactic acid), poly (methyl methacrylate), poly (vinylidene fluoride), respectively.

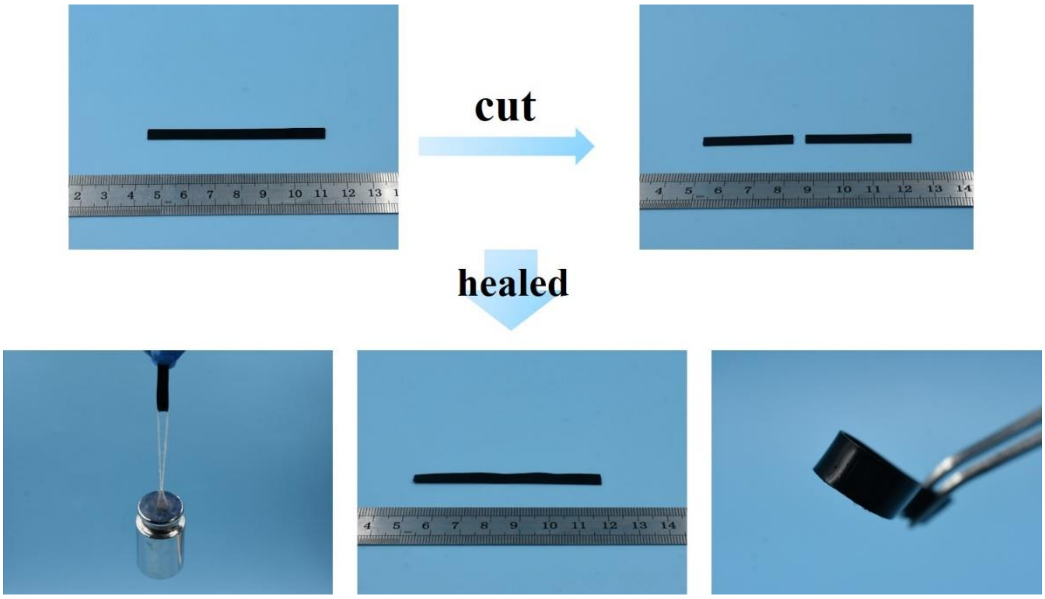

Fig. S3 Digital pictures of the healing process of the CG@CPA-7

Supplementary References

[S1] W. Xin, G.Q. Xi, W.T. Cao, C. Ma, T. Liu et al., Lightweight and flexible mxene/cnf/silver composite membranes with a brick-like structure and high-performance electromagnetic-interference shielding. RSC Adv. **9**(51), 29636-29644 (2019). https://doi.org/10.1039/c9ra06399d

[S2] L. Wang, H. Qiu, P. Song, Y. Zhang, Y. Lu et al., 3D $TI_3C_2T_x$ MXene/C hybrid foam/epoxy nanocomposites with superior electromagnetic interference shielding performances and robust mechanical properties. Compos. Part A-Appl. S **123**, 293-300 (2019). https://doi.org/10.1016/j.compositesa.2019.05.030

[S3] S. Pande, A. Chaudhary, D. Patel, B.P. Singh, R.B. Mathur, Mechanical and electrical properties of multiwall carbon nanotube/polycarbonate composites for electrostatic discharge and electromagnetic interference shielding applications. RSC Adv. **4**(27), 13839 (2014). https://doi.org/10.1039/c3ra47387b

[S4] M. Arjmand, T. Apperley, M. Okoniewski, U. Sundararaj, Comparative study of electromagnetic interference shielding properties of injection molded versus compression molded multi-walled carbon nanotube/polystyrene composites. Carbon. **50**(14), 5126-5134 (2012). https://doi.org/10.1016/j.carbon.2012.06.053

[S5] T. Kuang, L. Chang, F. Chen, Y. Sheng, D. Fu et al., Facile preparation of lightweight high-strength biodegradable polymer/multi-walled carbon nanotubes nanocomposite foams for electromagnetic interference shielding. Carbon **105**, 305-313 (2016). https://doi.org/10.1016/j.carbon.2016.04.052

[S6] M. Arjmand, M. Mahmoodi, G.A. Gelves, S. Park, U. Sundararaj, Electrical and electromagnetic interference shielding properties of flow-induced oriented carbon nanotubes in polycarbonate. Carbon **49**(11), 3430-3440 (2011). https://doi.org/10.1016/j.carbon.2011.04.039

[S7] Z. Liu, G. Bai, Y. Huang, Y. Ma, F. Du et al., Reflection and absorption contributions to the electromagnetic interference shielding of single-walled carbon nanotube/polyurethane composites. Carbon **45**(4), 821-827 (2007). https://doi.org/10.1016/j.carbon.2006.11.020

[S8] Y. Huang, N. Li, Y. Ma, D. Feng, F. Li et al., The influence of single-walled carbon nanotube structure on the electromagnetic interference shielding efficiency of its epoxy composites. Carbon **45**(8), 1614-1621 (2007). https://doi.org/10.1016/j.carbon.2007.04.016

[S9] N.C. Das, Y. Liu, K. Yang, W. Peng, S. Maiti et al., Single-walled carbon nanotube/poly(methyl methacrylate) composites for electromagnetic interference shielding. Polym. Eng Sci. **49**(8), 1627-1634 (2009). https://doi.org/10.1002/pen.21384

[S10] Y.L. Yang, M.C. Gupta. Novel carbon nanotube−polystyrene foam composites for electromagnetic interference shielding. Nano Lett. **5**(11), 2131-2134 (2005). http://dx.doi.org/10.1021/nl051375r

[S11] N. Li, Y. Huang, F. Du, X.B. He, X. Lin et al., Electromagnetic interference (EMI) shielding of single-walled carbon nanotube epoxy composites. Nano Lett. **6**(6), 1141-1145 (2006). http://dx.doi.org/10.1021/nl0602589

[S12] H. Wang, K. Zheng, X. Zhang, T. Du, C. Xiao et al., Segregated poly(vinylidene fluoride)/mwcnts composites for high-performance electromagnetic interference shielding. Compos. Part A-Appl. S. **90**, 606-613 (2016). https://doi.org/10.1016/j.compositesa.2016.08.030
